# Clinicopathologic and molecular characterization of a series of sporadic trichoblastic neoplasms

**DOI:** 10.1007/s00428-025-04335-8

**Published:** 2025-11-07

**Authors:** Carina A. Dehner, Eric C. Honaker, Asma K. Abu-Salah, Brandon A. Umphress, Rohini Mopuri, Numrah Fadra, Bryan Piatkowski, Rachel Kowal, Simon J. Warren, Ahmed Al-Omari, Ruifeng Guo

**Affiliations:** 1 Department of Pathology and Laboratory Medicine, University of Pennsylvania, 3400 Spruce Street, Philadelphia, PA 19104 USA; 2 Department of Pathology and Laboratory Medicine, Indiana University, Indianapolis, IN USA; 3 Department of Laboratory Medicine and Pathology, Mayo Clinic, Rochester, MN USA; 4 Department of Pathology, University of Michigan School of Medicine, Ann Arbor, MI USA; 5 Department of Laboratory Medicine and Pathology, Mayo Clinic, Jacksonville, FL USA

**Keywords:** Sporadic trichoblastic neoplasms, Trichogerminoma, Basaloid cells, Trichoblastoma

## Abstract

Trichoblastoma (TB) is a benign primitive follicular neoplasm that can occur in the setting of Brooke-Spiegler syndrome (*CYLD* mutations), in association with nevus sebaceous (mosaic *HRAS* mutations), or sporadically. We studied the histopathologic and molecular features of 16 sporadic trichoblastic neoplasms, including a case of trichogerminoma and a case of trichoblastic carcinoma arising within a TB. Sixteen tumors were identified in nine males and seven females (median age 64 years, range 33–97 years) involving the scalp (4), back (2), nasolabial fold (1), cheek (1), skin overlying the parotid gland (1), nasal ala (1), ear (1), upper chest (1), gluteal region (1), thigh (1), leg (1), and ankle (1) with a median size of 1.6 cm (range 1.2–7.0 cm). Histologically, 16 cases consisted of a dermal multinodular growth of basaloid epithelial cells surrounded by fibrotic stroma without epidermal connection. Malignant transformation was observed in one case, characterized by increased atypia and mitotic activity. Another case exhibited focal areas of “cell balls,” indicative of trichogerminoma. RNA sequencing of six tumors showed a high tumor mutational burden (TMB) and lacked a UV-related mutational signature, which may help distinguish trichoblastic tumors from potential mimics. Additionally, a *FOXK1::GRHL1* fusion was found in the case of trichogerminoma. Clinical follow-up (15/16 patients; 94%; median: 65 months; range 2.5–106.5 months) showed no evidence of residual or metastatic disease.

## Introduction

Trichoblastomas (TBs) are relatively rare cutaneous adnexal neoplasms arising from both follicular germinative cells and the surrounding fibrous root sheath [1]. Clinically, they are often slow-growing well-circumscribed nodules and show a predilection for the head and neck [2]. While these tumors can be part of the constellation of findings in Brooke-Spiegler syndrome or seen in association with nevus sebaceus, they also occur sporadically [3]. Morphologically, these tumors may resemble basal cell carcinoma due to their basaloid appearance and peripheral palisading [4–6]. However, they typically lack the mucinous stroma and artifactual clefting characteristic of basal cell carcinoma [4, 5]. Instead, they are often associated with a fibrotic stroma and may contain foci of papillary mesenchymal bodies [2, 4, 7]. Ultimately, histologic examination remains the diagnostic gold standard.

TBs are often benign; however, rare malignant transformation has been reported which may manifest de novo or as a trichoblastic carcinoma, trichoblastic sarcoma, or trichoblastic carcinosarcoma arising within a background TB [8, 9]. Benign TBs may show concerning features, such as deeply infiltrative growth or poor circumscription. These may be referred to as plaque-type TB or trichoblastic fibroma [10]. More specifically, these plaque-type variants have been shown to harbor somatic mutations in the Hedgehog pathway such as *PTCH*, although a prior study found that sporadic TBs lacked *PTCH* mutations [11, 12]. Additionally, a recent study on trichogerminoma showed evidence that these tumors were driven by *GRHL1/2/3* gene rearrangements [13]. Despite these findings, the molecular landscape of TBs remains poorly understood.

To further elucidate the molecular underpinnings of trichoblastic neoplasms, we performed a comprehensive clinicopathologic analysis of 16 cases, including RNA sequencing on a subset of six tumors.

## Materials and methods

This study was approved by the Institutional Review Board at the authors’ institutions. The cohort consisted of 16 cases of sporadic trichoblastic neoplasms which were retrieved from the internal archives and external consult services of the Indiana University School of Medicine and Mayo Clinic Department of Laboratory Medicine and Pathology by searching for “trichoblastoma/trichoblastic” with date restrictions from 2008 to 2023. Cases of tumors in the context of Brooke-Spiegler syndrome or those arising within a nevus sebaceous were excluded. All available hematoxylin and eosin (H&E) stained slides and immunohistochemical studies were reviewed by two dermatopathologists at the Indiana University School of Medicine with consensus on diagnosis and interpretation. Clinical information including age, gender, location, clinical follow-up, and molecular findings were obtained from the requisition and the electronic medical record system.

### Histologic examination

 Formalin-fixed paraffin-embedded (FFPE) tissue from each case was stained with hematoxylin and eosin (H&E). The diagnosis of trichoblastoma, trichogerminoma, and trichoblastic carcinoma was rendered following strict morphologic characteristics outlined in the literature. The identification of malignant transformation in trichoblastic carcinoma was based on increased atypia and mitotic activity within a background of “conventional” trichoblastoma.

### Immunohistochemistry

 Immunohistochemistry was performed on formalin-fixed paraffin-embedded tissue from one case (trichoblastic carcinoma) sectioned at 4 µm using the Dako Omnis instrument by Agilent Technologies Inc. (Santa Clara, CA) at the Indiana University Health Pathology Laboratory using antibodies against the following antigens: Ber-Ep4 (Dako, clone BerEp4, RTU), p63 (Dako, clone DAK-P63, RTU), CK20 (Dako, clone Ks20.8, RTU), Adipophilin (Dako, clone polyclonal, RTU), INSM1 (Dako, MRQ-70, RTU), SOX-10 (Dako, clone EP268, RTU), CEA (Dako, polyclonal, RTU), and EMA (Dako, clone E29, RTU) as well as Androgen receptor (Dako, clone SP107, RTU), c-MYB (Dako, clone Y69, RTU), S100 (Dako, polyclonal, RTU), Ki-67 (Dako, MIB-1, RTU), and p53 (Dako, clone DO-7, RTU).

### Molecular analysis

 RNA sequencing (RNAseq) was performed on six cases, including the case of trichogerminoma, at the Mayo Clinic Department of Laboratory Medicine and Pathology to assess for fusions, tumor mutational burden (TMB), and mutation signature.

MAPR-seq pipeline v3.1.4 was used for alignment against the human hg38 reference genome using the STAR aligner, and fusions were identified using the STAR-fusion caller. Fusions present in GTEX normal tissues and candidates with breakpoints less than 100 kb apart were excluded. Only potential fusion candidates determined to be in-frame with a cut-off of at least five total reads supporting the fusion (spanning reads and split reads) were included.

Putative somatic SNVs for TMB calculation were derived by applying five filters to RNAseq variants called using GATK HaplotypeCaller and scored with RV Boost. Five filters were applied: (1) SNVs (single nucleotide variation) supported by > 25 reads, RVBoost Qscore > 0.05, and genotype quality > 30 were retained; (2) SNVs with FFPE probability ≥ 0.5, as predicted by Excerno, were removed; (3) SNVs with allele frequencies between 0.45 and 0.55 or 0.95 and 1.0 were excluded; (4) variants present in dbSNP were removed; and (5) variants detected in GTEx skin samples were excluded. TMB was calculated using the following equation:

$$\mathrm{TMB}=\frac{\mathrm{Number\,of}\;\mathrm{somatic}\;\mathrm{mutations}\;(\mathrm{post}\;\mathrm{filter})}{\mathrm{Number}\;\mathrm{of}\;\mathrm{bases}\;\mathrm{covered}\geq50\mathrm x}\times1\times10^6$$

SNVs identified from the RNAseq data underwent analysis by the SigProfilerAssignment tool to identify and quantify the mutation signature. The tool assigns reference mutational signatures from Cosmic v3.3 to the extracted mutation signatures for a user-provided sample genome.

## Results

### Clinical features

 Sixteen tumors were identified which occurred in nine males (56.3%) and seven females (43.7%) with a median age of 64 years (range 33–97 years). Of these, 14 were diagnosed as “conventional” trichoblastomas with a slight male predominance (nine cases, 64.3%). The tumors were located on the scalp (4), back (2), nasolabial fold (1), cheek (1), skin overlying the parotid gland (1), nasal ala (1), ear (1), upper chest (1), leg (1), and ankle (1). Additionally, a single case of trichogerminoma was identified in the right medial thigh (1) of a male, and a single case of trichoblastic carcinoma was identified in the right gluteal region (1) of a female. The median size was 1.6 cm (range 1.2–7.0 cm). Clinical follow-up data were available for 15 of 16 patients (94%), with a median duration of 65 months (range 2.5–106.5 months). At the time of last follow-up, 12 patients (80%) were disease-free, while three patients (20%) had died from unrelated causes, including metastatic breast cancer and lung cancer (Table 1).

**Table 1:** Comprehensive clinicopathologic findings in 16 cases of trichoblastic tumors, including a single case of trichogerminoma (*) and a single case of trichoblastic carcinoma (**)

Case no	Specimen type	Age (years)	Sex	Tumor location	Molecular findings	Follow-up duration (months)	Disease status at follow-up	Cause of death (if applicable)
1*	Excision	62	M	Right medial thigh	*FOXK1::GRHL1* fusion, high TMB	47	No evidence of disease	—
2	Shave biopsy	65	F	Left nasolabial fold	High TMB	59	No evidence of disease	—
3	Shave biopsy	81	M	Left upper chest	High TMB	45	No evidence of disease	—
4	Excision	95	F	Right leg	High TMB	37	Disease-free	Metastatic breast cancer
5	Excision	33	F	Left cheek	High TMB	78	No evidence of disease	—
6	Shave biopsy	97	M	Right nasal ala	High TMB	84	Disease-free	Unknown
7	Excision	71	M	Scalp	—	Lost to follow-up	—	—
8**	Re-excision	56	F	Right gluteal mass	—	2.5	No evidence of disease	—
9	Excision	44	M	Back-thoracic region	—	78	No evidence of disease	—
10	Excision	39	M	Back	—	106.5	No evidence of disease	—
11	Excision	72	F	Right upper lip	—	65	Disease-free	Lung cancer
12	Excision	78	F	Crown of scalp	—	96	No evidence of disease	—
13	Excision	83	F	Apex of scalp	—	46	No evidence of disease	—
14	Excision	57	M	Skin overlying parotid gland	—	102	No evidence of disease	—
15	Shave biopsy	48	M	Right scalp	—	27	No evidence of disease	—
16	Excision	59	M	Left medial ankle	—	97	No evidence of disease	—

*TMB* tumor mutation burden, *MS* mutation signature

### Morphologic features

**Fig. 1:**
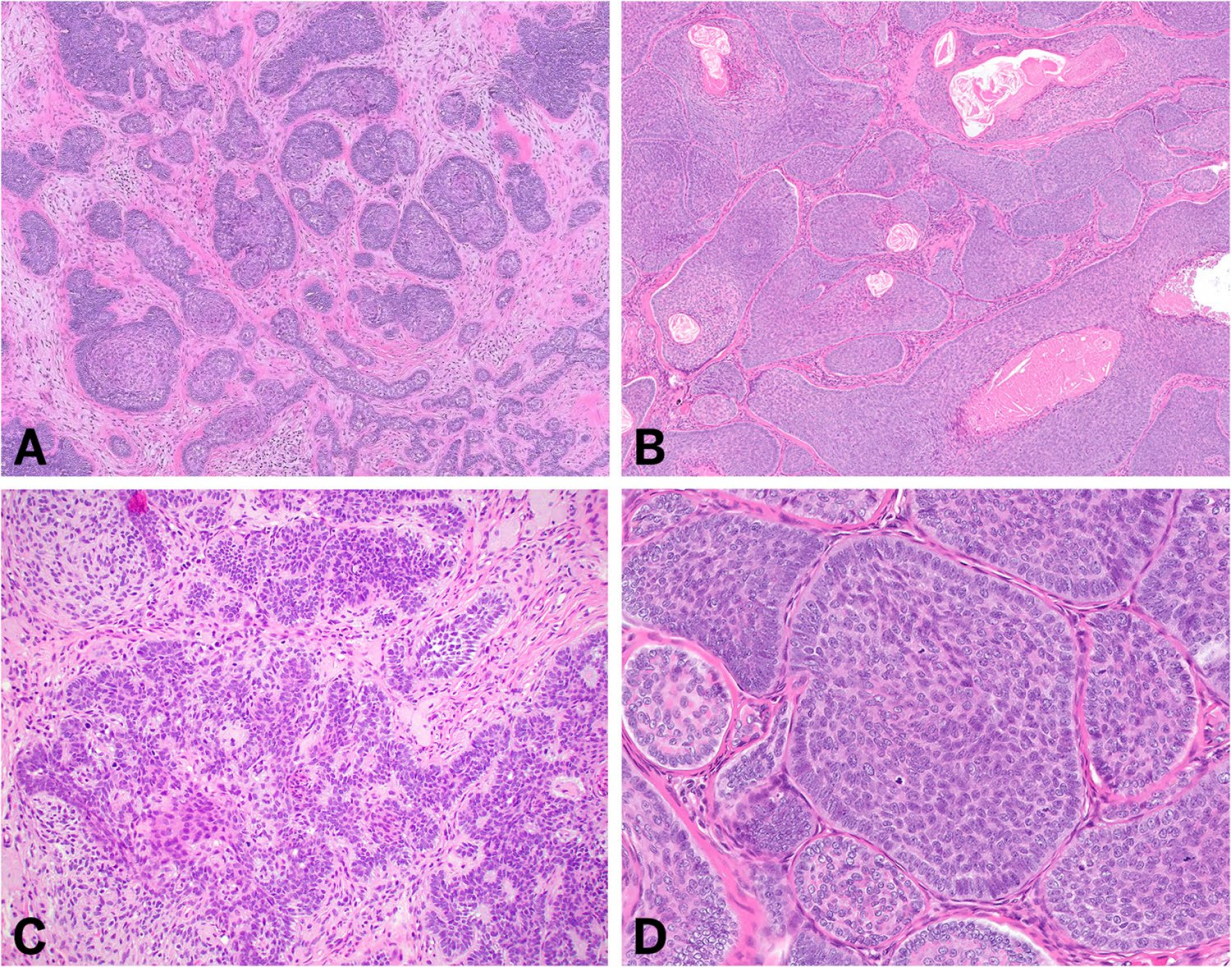
Histologic features of “conventional” trichoblastoma. Low magnification (**A**–**B**) hematoxylin and eosin-stained sections shows a multinodular proliferation of lesion cells arranged in nests surrounded by a dense cellular and fibrotic stroma with occasional keratin cysts. High magnification (**C**–**D**) hematoxylin and eosin-stained sections demonstrates nests of basaloid cells with rare pseudoglandular formation

 Fourteen of 16 cases demonstrated similar histologic features characterized by a predominately lobular architecture with a dermal, multinodular follicular proliferation of basaloid epithelial cells arranged in nests and strands surrounded by a dense cellular and fibrotic stroma (Fig. 1). Occasional keratin cysts, rare papillary mesenchymal bodies, and focal pseudoglandular formation associated with mucin deposition were observed. In all cases, the epidermis was uninvolved.

**Fig. 2:**
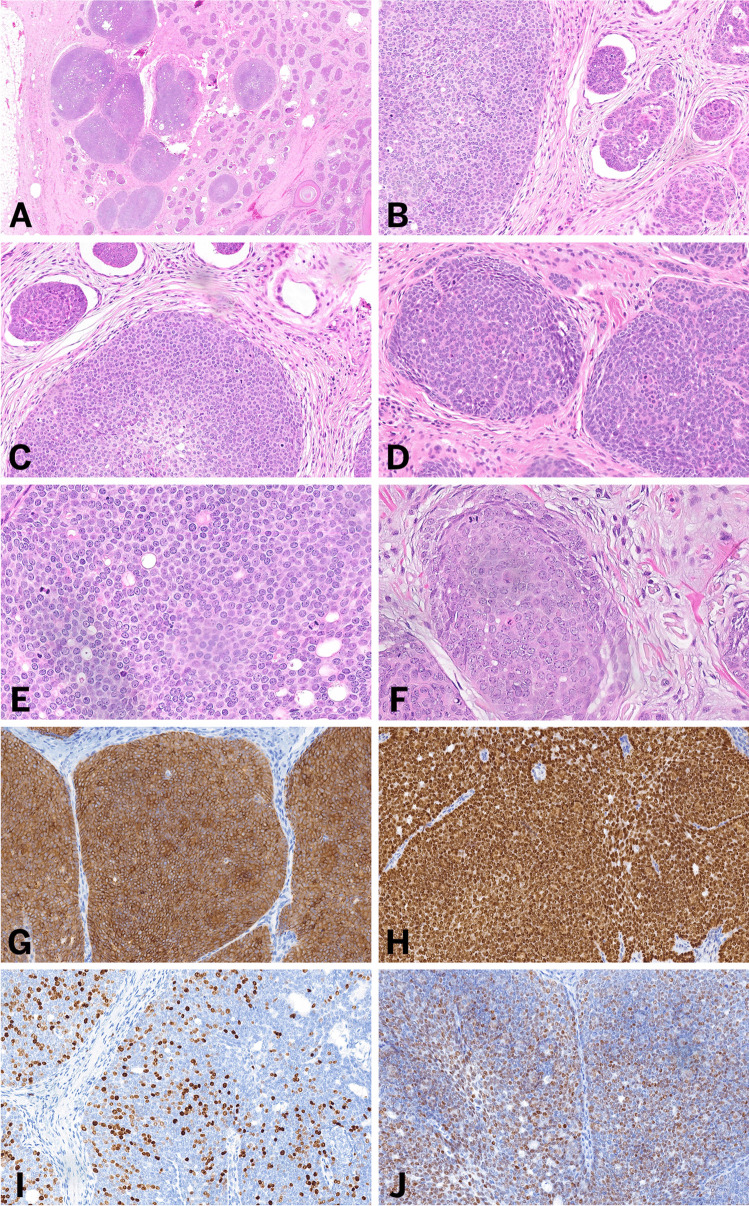
Histologic features of trichoblastic carcinoma. Low magnification (**A**) hematoxylin and eosin-stained sections shows similar findings to Fig. 1, including a dermal proliferation of basaloid cells arranged in nests. However, low (**B**) and high magnification (**C**) hematoxylin and eosin-stained sections show a clear demarcation between malignant nests and benign trichoblastoma components. On high magnification of the malignant nests (**D**–**F**), nuclear atypia, increased mitotic activity, and apoptosis are observed. Immunohistochemical studies demonstrate that the nests of atypical cells show diffuse expression of BerEP4 (**G**) and p63 (**H**), as well as a high Ki-67 proliferative index (**I**) and increased p53 expression (**J**)

One case demonstrated large, circumscribed aggregates of cytologically atypical basaloid cells with frequent mitotic figures and abundant apoptosis (Fig. 2). These aggregates involved the full thickness of the dermis and extended into the subcutaneous fat. Within the background, smaller nests and strands of basaloid epithelial cells with benign cytologic features, morphologically similar to the 14 cases of “conventional” trichoblastoma described above, were observed. These findings were consistent with trichoblastic carcinoma arising in the setting of a “conventional” trichoblastoma.

**Fig. 3:**
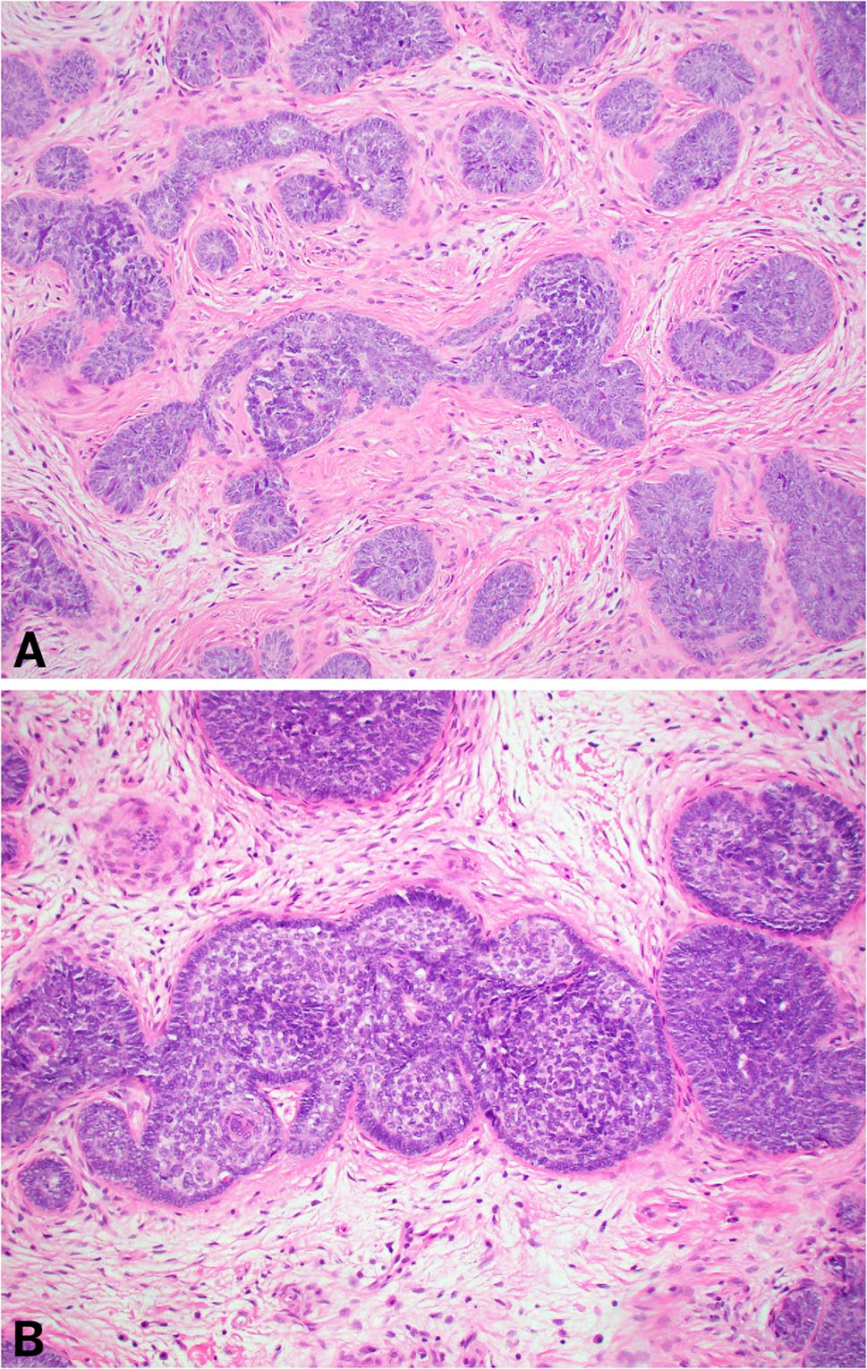
Histologic features of trichogerminoma. Low magnification (**A**–**B**) hematoxylin and eosin-stained sections show tightly packed round clusters of basaloid cells (“cell balls”) with peripheral palisading and a background of fibrous and myxoid stroma

Another case demonstrated focal areas of tightly packed round clusters of basaloid cells (“cell balls”) with peripheral palisading with a background of fibrous and myxoid stroma, consistent with trichogerminoma-like morphology (Fig. 3).

### Immunohistochemical features

 Six cases of conventional trichoblastoma were tested for CK20 expression and five of six cases showed a few isolated CK20-positive cells, consistent with the expected staining pattern.

In a single case of trichoblastic carcinoma, immunohistochemical analysis revealed diffuse cytoplasmic Ber-Ep4 and p63 reactivity. The tumor cells were negative for CK20, adipophilin, INSM1, SOX-10, CEA, EMA, androgen receptor (AR), c-MYB, and S100. The Ki-67 proliferative index was elevated, reaching up to 60% within larger basaloid aggregates, highlighting increased cellular proliferation. Additionally, p53 expression was notably increased in the large aggregates of lesional cells.

### Molecular findings

**Fig. 4:**
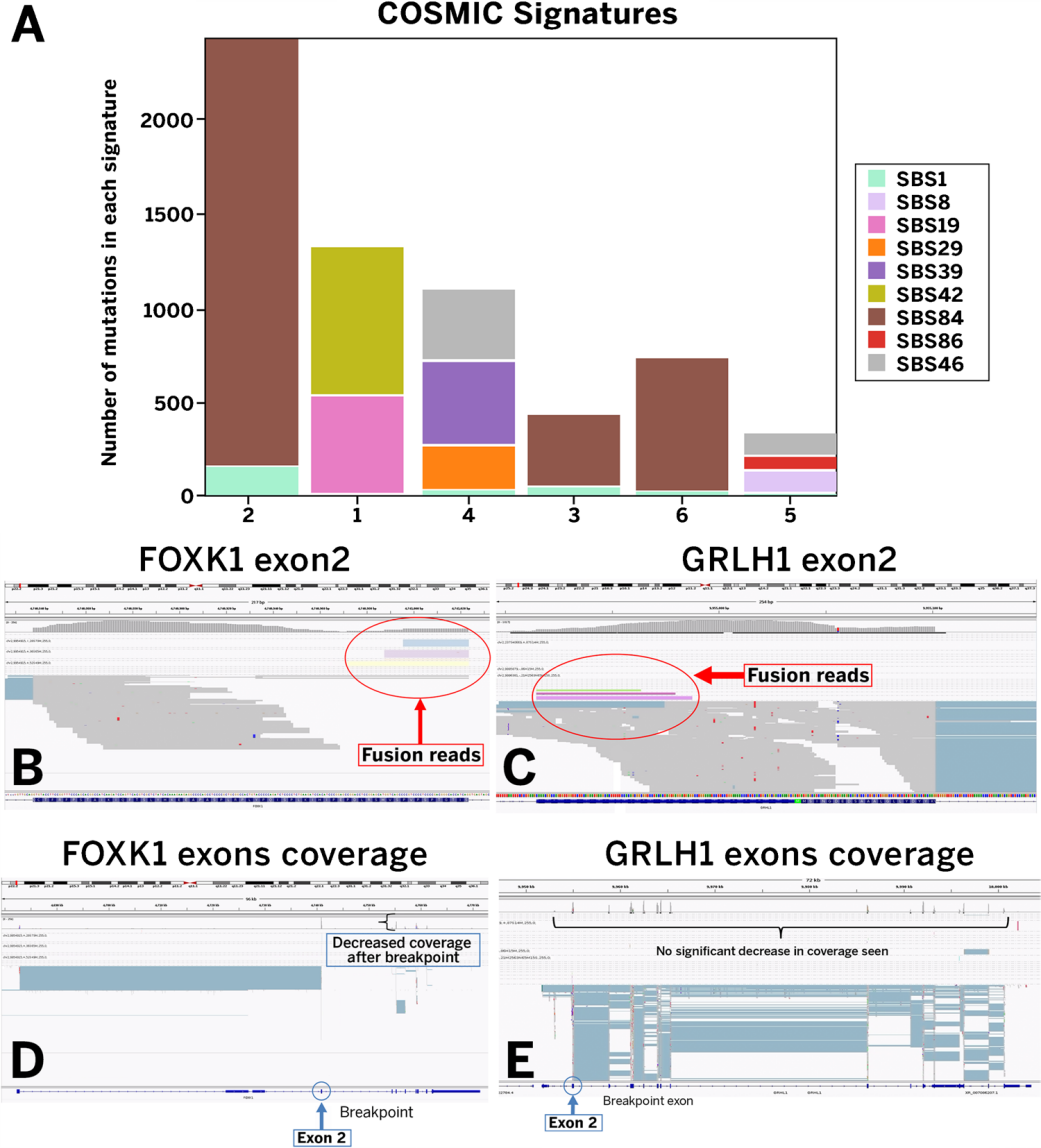
Molecular findings of the studied cases. **A** Cosmic signatures per individual tested case. The *x*-axis indicates the respective case number. **B** Fusion diagram of the *FOXKR1::GRLH1* fusion identified in the case of trichogerminoma

 RNA sequencing identified a *FOXK1*(chr7 exon 2)*::GRHL1*(chr2 exon 2) fusion in the case of trichogerminoma, accompanied by decreased *FOXK1* expression downstream of the fusion breakpoint. Additionally, several *MALAT1* fusions were identified with *MALAT1* as the 3′ partner and 5′ partner (Fig. 4).

All cases that underwent RNA sequencing (6/6, 100%) demonstrated a high TMB. Mutation signature analysis, using COSMIC mutation signatures as a background, revealed universal (6/6) presence of SBS1 (albeit a small number in a subset of cases), substitutions resulting from 5-methylcytosine deamination. The second most prevalent signature, SBS84, attributed to activation-induced cytidine deaminase (AID) activity, was observed in 50% of cases. Interestingly, SBS38, the COSMIC signature most commonly associated with UV-light damage, was uniquely absent in all six tested samples.

## Discussion

Trichoblastomas (TB) are relatively uncommon cutaneous adnexal neoplasms of follicular origin with a predilection for the head and neck region. These tumors may arise in the setting of Brooke-Spiegler [3–16], or Rombo syndrome [17, 18], in which case they are most commonly seen in young female patients [16], or they may arise sporadically in middle-aged to older adults [17]. Genetically, *CYLD* mutations are found in syndromic settings, while sporadic cases may show mutations involving *HRAS*, especially if arising in the background of nevus sebaceous [3]. One of the major challenges clinically and pathologically is that there is significant overlap between TB and other basaloid neoplasms, such as basal cell carcinoma (BCC).

Several previous studies have investigated the immunohistochemical phenotype of TB and trichoblastic carcinoma to aid in differentiating them from histologic mimics [5, 9, 19]. While not shown in the present study, trichoblastic tumors have been shown to typically be Ber-EP4 and AR negative [4]. However, rare exceptions exist as demonstrated in the present study which identified a trichoblastic carcinoma with diffuse Ber-EP4 expression. Additionally, trichoblastic tumors have been reported to show focal and sparse CK20 reactivity in Merkel cells [4, 5]. CD10 reactivity within surrounding stroma is also supportive [5, 9, 19]. In the present case of trichoblastic carcinoma, increased p53 expression and Ki-67 index were observed. However, given the absence of comparative staining in the benign trichoblastomas within this cohort and the lack of evidence on this topic in the literature, these findings should be interpreted with caution until further research identifies the diagnostic utility of this observation.

Just like many other mimics of BCC, trichoblastic tumors may originally be misdiagnosed. It is accepted that BCC is typically driven by alterations impacting the Sonic Hedgehog (Hh) pathway (i.e. *PTCH1* and *TP53*) and often harbors hallmark ultraviolet (UV)-related mutation signatures, including C > T transitions at dipyrimidine sites [20, 21]. While TBs usually lack mutations involving *PTCH1*, to the best of our knowledge, comprehensive molecular studies focusing on sporadic TB have not been conducted. Our study identified a possible unique aberrant molecular pattern which has the potential to serve as a diagnostic tool in delineating between these two entities. Specifically, RNA sequencing of six cases of TB identified a universal high TMB and a mutational signature of SBS1, substitutions resulting from 5-methylcytosine deamination. While this may not be entirely specific, it is noteworthy that none of the cases harbored a known UV-related mutational signature. Thus, in the appropriate clinical and histologic context, the absence of UV-related mutational signatures may provide supportive molecular evidence for trichoblastic tumors and serve as a distinguishing feature from BCC. However, due to the limitation of RNA sequencing in detecting mutational signatures (see below), this finding requires further investigation to establish its legitimacy. Ultimately, evaluating a combination of morphology, immunohistochemistry, and the presence or absence of molecular aberrations is most helpful when differentiating between trichoblastic tumors and BCC (see Table 2).

**Table 2:** A comparison of the morphologic, immunohistochemical, and molecular features of trichoblastoma, trichogerminoma, trichoblastic carcinoma, and basal cell carcinoma

Feature	Trichoblastoma (TB)	Trichogerminoma	Trichoblastic carcinoma	Basal cell carcinoma (BCC)
Morphology	Multinodular follicular proliferation of basaloid epithelial cells, papillary mesenchymal bodies, keratin cysts	Lobules of basaloid cells with central “cell balls” and peripheral palisading	Basaloid nests with atypia, mitoses, apoptosis, necrosis; may have adjacent benign TB component	Basaloid nests; peripheral palisading; clefting; mucinous stroma, vary based on subtype
Location	Dermis to subcutis	Dermis to subcutis	Dermis to subcutis	Epidermis to dermis
Margins	Pushing	Pushing	Infiltrative	Infiltrative
Necrosis	Absent	Often absent	Present	May be present
Immunohistochemistry	CK20 + Merkel cells (retained); CD10 + (stromal); BerEP4-(typically); AR-	CK5/6 +; P63 +; CD10 + (stromal); Bcl-2 + (ring-like peripheral rim of “cell balls”)	Variable; often show high Ki-67	BerEP4 +; CD10 + (epithelial); AR ±; CK20 −
Molecular features	*CYLD* mutations in syndromic cases; may be negative for UV-related* mutation signatures; possible fusions: *YAP1::MAML2*, *YAP1::NUTM1*, and *RNF13::PAK2*	Existing data suggests fusions involving *GRHL1/2/3*, such as *FOXK1*::*GRHL1*	May show p53 and PI3-AKT aberrations	*PTCH1* mutations common; Hedgehog pathway activation; often show UV-related* mutation signatures
Clinical behavior	Benign	Benign	Malignant	Locally aggressive; rarely metastasizes
Associated syndromes	Nevus sebaceus; Brooke-Spiegler (*CYLD*)	None currently known	May arise from TB or de novo	Gorlin syndrome (*PTCH1*)

As stated above, all cases of trichoblastoma which underwent RNA sequencing exhibited a mutational signature of SBS1. Previous reports have shown that the number of SBS1 mutations correlates with cell age and type, as well as may reflect the number of mitoses a cell has undergone in its lifetime [22, 23]. The second most prevalent signature in 50% of cases, SBS84, has been previously reported in lymphoid neoplasms [22]. Notably, a recent case series by Kervarrec et al. identified *YAP1::MAML2*, *YAP1::NUTM1*, and *RNF13::PAK2* in-frame fusions in four cases of benign TB as well as other adnexal neoplasms, suggesting possible oncogenic drivers [24]. Although both, the present study and the study by Kervarrec et al., employed RNA sequencing to detect gene alterations, no recurrent or recognized fusion was detected in our analysis. This discrepancy may be due to many factors, including the technical limitations of RNA sequencing on formalin-fixed, paraffin-embedded tissue (outlined below), primer-related limitations, as well as the potential biological heterogeneity of trichoblastomas, which may be driven by a diverse range of oncogenic mechanisms beyond gene fusions [25].

We also reported one case of trichoblastic carcinoma, which may arise de novo or within an existing benign TB [8, 9, 25–29]. Malignant transformation has been described in sporadic and syndromic (i.e., *CYLD* mutation) trichoblastomas [26, 29–33]. The diagnosis of malignant transformation relies on the identification of cytologic atypia and mitotic activity within basaloid tumor nests with or without apoptosis and necrosis [8, 9, 25–27]. These tumors have been stratified into low-grade and high-grade categories with the low-grade variants demonstrating more indolent behavior, while their high-grade counterparts are associated with a poor prognosis [26, 29]. A more recent investigation, however, has suggested that these tumors may be less aggressive than previously thought [27]. The pathogenesis of malignant transformation to trichoblastic carcinoma remains poorly understood, although p53 and PI3-AKT aberrations are thought to possibly play a role [8, 28]. While we believe that it would be highly informative to pursue molecular testing on the precursor and malignant components, regrettably, we were unable to perform additional testing in our case.

We herein also identified a case of trichogerminoma, often referred to as the trichogerminoma variant of TB, which is a distinct adnexal follicular neoplasm that exhibits a characteristic histologic appearance of well-circumscribed nodules of densely packed rounded nests of basaloid cells forming “cell balls” with variably cellular stroma [13, 33–38]. Given the distinct and characteristic morphologic and molecular features of trichogerminoma, this may indeed be a unique entity and not a “true” variant of TB. Malignant transformation of a trichogerminoma is exceedingly rare, with only two cases reported in literature [39]. Recurrent *FOXK1*::*GRHL1* fusions have been identified in a previous case series of trichogerminomas by Kervarrec et al. which also share the histologic manifestations of the present case [13]. The clinical significance of this fusion remains unclear; however, it is hypothesized that fusions involving *GRHL1/2/3* may be the oncogenic driver and identification of such may represent a helpful diagnostic tool to separate it from other morphologic mimics.

*FOXK1* (forkhead box K1) is localized to chromosome 7p22.1 and is involved in many physiologic processes including regulation of metabolism (i.e., aerobic glycolysis), autophagy, and cell differentiation, migration, and proliferation [40, 41]. *FOXK1*-related fusions have been identified in previous reports of trichogerminoma [13], malignant melanoma [42, 43], adenocarcinoma of the prostate [42], ovary [42], and breast [43].

In contrast, the fusion partner *GRHL1* is located on chromosome 2p25.1 and encodes grainyhead like transcription factor 1 which is involved in human development [44]. Fusions involving *GRHL1* have been identified in previous reports of trichogerminomas [13] and sebaceomas [45].

As a case series, there are several limitations to the present study. Most notably, while RNA sequencing provides insight into expressed gene fusions and potential somatic variants, there are many limitations in its use for analyzing TMB and mutational signatures [46]. Unlike DNA-based methods, RNA sequencing has reduced sensitivity and specificity for detecting single nucleotide variants and small insertions/deletions (indels), particularly in low-expression or non-expressed genes, as it has a bias toward highly expressed genes [46]. Furthermore, without matched normal tissue, distinguishing somatic mutations from germline variants with RNA sequencing remains challenging and may lead to an overestimation of TMB due to residual germline contamination and technical artifacts, even after stringent filtering [46, 47]. Similarly, while mutational signature analysis from RNAseq data is feasible, it remains less robust compared to DNA-derived signatures, especially for detecting UV-related patterns, which may be underrepresented or missed if the relevant genes are not expressed at sufficient levels. This is a key limiting point of the present study, as we highlight the lack of UV-related signature in TB as a diagnostic tool when trying to exclude BCC. Given these limitations, our observations regarding high TMB, the presence of SBS1 and SBS84 mutational signatures, and the absence of a UV-related mutational signature should be interpreted as preliminary until further validation using DNA-based sequencing with matched normal tissue is performed to confirm these findings and assess their clinical relevance.

In conclusion, trichoblastoma is a rare cutaneous adnexal neoplasm composed of nested basaloid cells that may undergo malignant transformation. Trichogerminoma, traditionally thought to be a variant of trichoblastoma, has been shown to commonly harbor *GRHL1/2/3*-related fusions. While immunohistochemistry can aid in distinguishing trichoblastic tumors from other cutaneous basaloid entities, its utility remains limited. While additional studies with larger case numbers and additional comprehensive molecular analyses are needed, our study provides evidence that a non-UV-related mutational signature, specifically SBS1, may serve as a helpful diagnostic feature in differentiating basal cell carcinoma from trichoblastic tumors.

## Data Availability

Data can be made available upon reasonable request.

## References

[1] Headington JT (1970) Differentiating neoplasms of hair germ. J Clin Pathol 23(6):464–471. 10.1136/jcp.23.6.464 5476873 10.1136/jcp.23.6.464 PMC476809

[2] Ghigliotti G, De Col E, Parodi A, Bombonato C, Argenziano G (2016) Trichoblastoma: is a clinical or dermoscopic diagnosis possible? J Eur Acad Dermatol Venereol 30(11):1978–1980. 10.1111/jdv.13830 27439654 10.1111/jdv.13830

[3] Jaqueti G, Requena L, Yus ES (2000) Trichoblastoma is the most common neoplasm developed in nevus sebaceus of Jadassohn. Am J Dermatopathol 22(2):108–118. 10.1097/00000372-200004000-00004 10770429 10.1097/00000372-200004000-00004

[4] Patel P, Nawrocki S, Hinther K, Khachemoune A (2020) Trichoblastomas mimicking basal cell carcinoma: the importance of identification and differentiation. Cureus 12(5):e8272. 10.7759/cureus.8272 32596088 10.7759/cureus.8272 PMC7314372

[5] Stanoszek LM, Wang GY, Harms PW (2017) Histologic mimics of basal cell carcinoma. Arch Pathol Lab Med 141(11):1490–1502. 10.5858/arpa.2017-0222-RA 29072946 10.5858/arpa.2017-0222-RA

[6] Tellechea O, Cardoso JC, Reis JP et al (2015) Benign follicular tumors. An Bras Dermatol 90(6):780–798. 10.1590/abd1806-4841.20154114 26734858 10.1590/abd1806-4841.20154114 PMC4689065

[7] Woods AD, Grushchak S, Rakita U, Liu W, Petronic-Rosic V, Krunic AL (2023) Plaque variant trichoblastoma—an unusually aggressive neoplasm: presentation of 11 cases in 4 individuals and a review of the literature. JAAD Case Rep 36:108–112. 10.1016/j.jdcr.2023.04.021 37288444 10.1016/j.jdcr.2023.04.021 PMC10242479

[8] Boettler MA, Shahwan KT, Abidi NY, Carr DR (2022) Trichoblastic carcinoma: a comprehensive review of the literature. Arch Dermatol Res 314(5):399–403. 10.1007/s00403-021-02241-y 33993349 10.1007/s00403-021-02241-y

[9] Rance LB, Fraga GR (2024) A diagnosis of trichoblastic carcinoma using immunohistochemistry. Kans J Med 17(6):153–155. 10.17161/kjm.vol17.22437 39758540 10.17161/kjm.vol17.22437 PMC11698578

[10] Altman DA, Mikhail GR, Johnson TM, Lowe L (1995) Trichoblastic fibroma. A series of 10 cases with report of a new plaque variant. Arch Dermatol 131(2):198–201. 10.1001/archderm.131.2.198 7857118 10.1001/archderm.131.2.198

[11] Hafner C, Schmiemann V, Ruetten A et al (2007) PTCH mutations are not mainly involved in the pathogenesis of sporadic trichoblastomas. Hum Pathol 38(10):1496–1500. 10.1016/j.humpath.2007.02.015 17597182 10.1016/j.humpath.2007.02.015

[12] Giang J, Mooyaart AL, Martens-de Kemp SR et al (2023) Hedgehog pathway mutations are involved in the pathogenesis of plaque-type “trichoblastoma”: a report of two cases. J Cutan Pathol 50(7):674–680. 10.1111/cup.14389 36607280 10.1111/cup.14389

[13] Kervarrec T, Pissaloux D, Poilane J et al (2022) Recurrent FOXK1::GRHL and GPS2::GRHL fusions in trichogerminoma. J Pathol 257(1):96–108. 10.1002/path.5872 35049062 10.1002/path.5872

[14] Kazakov DV (2016) Brooke-Spiegler syndrome and phenotypic variants: an update. Head Neck Pathol 10(2):125–130. 10.1007/s12105-016-0705-x 26971504 10.1007/s12105-016-0705-x PMC4838966

[15] Manchanda K, Bansal M, Bhayana AA, Pandey S (2012) Brooke-Spiegler syndrome: a rare entity. Int J Trichol 4(1):29–31. 10.4103/0974-7753.96084 10.4103/0974-7753.96084 PMC3358935 22628987

[16] Schukow C, Ahmed A (2023) Trichoblastoma and Trichoepithelioma. StatPearls Publishing

[17] Ashinoff R, Jacobson M, Belsito DV (1993) Rombo syndrome: a second case report and review. J Am Acad Dermatol 28(6):1011–1014. 10.1016/S0190-9622(08)80656-1 8496444 10.1016/s0190-9622(08)80656-1

[18] Michaëlsson G, Olsson E, Westermark P (1981) The rombo syndrome: a familial disorder with vermiculate atrophoderma, milia, hypotrichosis, trichoepitheliomas, basal cell carcinomas and peripheral vasodilation with cyanosis. Acta Derm Venereol 61(6):497–503 6177160

[19] Alomari A, Subtil A, Owen CE, McNiff JM (2013) Solitary and multiple tumors of follicular infundibulum: a review of 168 cases with emphasis on staining patterns and clinical variants. J Cutan Pathol 40(6):532–537. 10.1111/cup.12137 23531053 10.1111/cup.12137

[20] Bonilla X, Parmentier L, King B et al (2016) Genomic analysis identifies new drivers and progression pathways in skin basal cell carcinoma. Nat Genet 48(4):398–406. 10.1038/ng.3525 26950094 10.1038/ng.3525

[21] Kilgour JM, Jia JL, Sarin KY (2021) Review of the molecular genetics of basal cell carcinoma; inherited susceptibility, somatic mutations, and targeted therapeutics. Cancers (Basel). 10.3390/cancers13153870 34359772 10.3390/cancers13153870 PMC8345475

[22] Alexandrov LB, Kim J, Haradhvala NJ et al (2020) The repertoire of mutational signatures in human cancer. Nature 578(7793):94–101. 10.1038/s41586-020-1943-3 32025018 10.1038/s41586-020-1943-3 PMC7054213

[23] Alexandrov LB, Jones PH, Wedge DC et al (2015) Clock-like mutational processes in human somatic cells. Nat Genet 47(12):1402–1407. 10.1038/ng.3441 26551669 10.1038/ng.3441 PMC4783858

[24] Kervarrec T, Macagno N, Houlier A et al (2025) YAP1::MAML2, YAP1::NUTM1, and RNF13::PAK2 rearrangements in trichoblastomas and adnexal tumors with panfollicular differentiation: expanding the spectrum of YAP1/PAK-fused skin adnexal tumors. Virchows Arch. 10.1007/s00428-025-04175-6 40689944 10.1007/s00428-025-04175-6

[25] Lee JS, Kwon JH, Jung GS et al (2018) A giant trichoblastic carcinoma. Arch Craniofac Surg 19(4):275–278. 10.7181/acfs.2018.02124 30613089 10.7181/acfs.2018.02124 PMC6325323

[26] Yaacoub E, El Borgi J, Challita R, Sleiman Z, Ghanime G (2020) Pinna high grade trichoblastic carcinoma: a report. Clin Pract 10(3):1204. 10.4081/cp.2020.1204 32952982 10.4081/cp.2020.1204 PMC7482185

[27] Mehta A, Davey J, Wiedemeyer K, Brenn T (2021) Morphologically high-grade trichoblastic carcinoma: a clinicopathological study with long-term clinical follow-up. Histopathology 78(7):970–975. 10.1111/his.14325 33393120 10.1111/his.14325

[28] Mouchard A, Monegier-Dusorbier C, Berthon P et al (2022) High-grade trichoblastic carcinoma with sarcomatoid differentiation harboring TP53 and PIK3CA mutations. Ann Dermatol Venereol 149(1):74–77. 10.1016/j.annder.2021.07.006 34838338 10.1016/j.annder.2021.07.006

[29] Thomas M, Bruant-Rodier C, Bodin F, Cribier B, Huther M, Dissaux C (2017) De l’intérêt de différencier les carcinomes trichoblastiques (CT) des carcinomes basocellulaires (CBC). À propos de 21 cas [Why is it important to differentiate trichoblastic carcinomas (CT) from basal cell carcinomas (CBC). About 21 cases]. Annales de Chirurgie Plastique Esthétique 62(3):212–218. 10.1016/j.anplas.2017.03.001 10.1016/j.anplas.2017.03.001 28385570

[30] Schulz T, Proske S, Hartschuh W, Kurzen H, Paul E, Wunsch PH (2005) High-grade trichoblastic carcinoma arising in trichoblastoma. Am J Dermatopathol 27(1):9–16. 10.1097/01.dad.0000142240.93956.cb 15677970 10.1097/01.dad.0000142240.93956.cb

[31] Janeczek M, Lehrer M, Pockaj B, DiCaudo D, Ochoa S (2021) Metastatic trichoblastic carcinoma in the setting of trichoblastomatosis and multiple facial trichoepitheliomas. JAAD Case Rep 16:127–129. 10.1016/j.jdcr.2021.08.024 34584923 10.1016/j.jdcr.2021.08.024 PMC8455310

[32] Fusumae T, Tanese K, Takeuchi A et al (2019) High-grade trichoblastic carcinoma arising through malignant transformation of trichoblastoma: immunohistochemical analysis and the expression of p53 and phosphorylated AKT. J Dermatol 46(1):57–60. 10.1111/1346-8138.14686 30379345 10.1111/1346-8138.14686

[33] Chen LL, Hu JT, Li Y (2013) Trichogerminoma, a rare cutaneous follicular neoplasm with indolent clinical course: report of two cases and review of literature. Diagn Pathol 8(1):210. 10.1186/1746-1596-8-210 24354761 10.1186/1746-1596-8-210 PMC3878257

[34] Sau P, Lupton GP, Graham JH (1992) Trichogerminoma. J Cutan Pathol 19(5):357–365. 10.1111/j.1600-0560.1992.tb00606.x 1282133 10.1111/j.1600-0560.1992.tb00606.x

[35] Pozo L, Diaz-Cano SJ (2005) Trichogerminoma: further evidence to support a specific follicular neoplasm. Histopathology 46(1):108–110. 10.1111/j.1365-2559.2005.01955.x 15656895 10.1111/j.1365-2559.2005.01955.x

[36] Tellechea O, Reis JP (2009) Trichogerminoma. Am J Dermatopathol 31(5):480–483. 10.1097/DAD.0b013e31818ffcc3 19542926 10.1097/DAD.0b013e31818ffcc3

[37] Shin JH, Jung JH, Yoo J, Kang J, Lee KY (2007) Pigmented trichogerminoma-a case report. Korean J Pathol 41:187–196

[38] Kazakov DV, Kutzner H, Rütten A, Dummer R, Burg G, Kempf W (2002) Trichogerminoma: a rare cutaneous adnexal tumor with differentiation toward the hair germ epithelium. Dermatology 205(4):405–408. 10.1159/000066427 12444341 10.1159/000066427

[39] Kervarrec T, Mancini M, Tallet A, Mourah S, Battistella M, Frouin E (2025) Trichogerminoma with malignant transformation. Virchows Arch 487(4):909. 10.1007/s00428-025-04032-6 39853341 10.1007/s00428-025-04032-6

[40] National Center for Biotechnology Information (NCBI). FOXK1 forkhead box K1 [Homo sapiens (human)]. www.ncbi.nlm.nih.gov. Accessed 08 June 2025

[41] Garry DJ, Maeng G, Garry MG (2020) Foxk1 regulates cancer progression. Ann Transl Med 8(17):1041–1041. 10.21037/atm-2020-94 33145260 10.21037/atm-2020-94 PMC7575999

[42] Gao Q, Liang WW, Foltz SM et al (2018) Driver fusions and their implications in the development and treatment of human cancers. Cell Rep 23(1):227-238.e3. 10.1016/j.celrep.2018.03.050 29617662 10.1016/j.celrep.2018.03.050 PMC5916809

[43] Hu X, Wang Q, Tang M et al (2018) Tumorfusions: an integrative resource for cancer-associated transcript fusions. Nucleic Acids Res 46(D1):D1144–D1149. 10.1093/nar/gkx1018 29099951 10.1093/nar/gkx1018 PMC5753333

[44] National Center for Biotechnology Information (NCBI). GRHL1 grainyhead like transcription factor 1 [Homo sapiens (human)]. www.ncbi.nlm.nih.gov. Accessed 08 June 2025

[45] Legrand M, Louveau B, Macagno N et al (2025) Recurrent GRHL fusions in a subset of sebaceoma: microscopic and molecular characterisation of eight cases. Histopathology 86(4):571–584. 10.1111/his.15361 39564735 10.1111/his.15361 PMC11791738

[46] Coudray A, Battenhouse AM, Bucher P, Iyer VR (2018) Detection and benchmarking of somatic mutations in cancer genomes using RNA-seq data. PeerJ 6:e5362. 10.7717/peerj.5362 30083469 10.7717/peerj.5362 PMC6074801

[47] Zhang J, Baran J, Cros A et al (2011) International cancer genome consortium data portal–a one-stop shop for cancer genomics data. Database (Oxford) 2011(0):bar026. 10.1093/database/bar026 21930502 10.1093/database/bar026 PMC3263593

